# Primer on Precision Medicine for Complex Chronic Disorders

**DOI:** 10.14309/ctg.0000000000000067

**Published:** 2019-07-22

**Authors:** David C. Whitcomb

**Affiliations:** 1 Department of Medicine, Cell Biology and Molecular Physiology, and Human Genetics, University of Pittsburgh, Pittsburgh, Pennsylvania, USA.

## Abstract

Precision medicine promises patients with complex disorders the right treatment for the right patient at the right dose at the right time with expectation of better health at a lower cost. The demand for precision medicine highlights the limitations of modern Western medicine. Modern Western medicine is a population-based, top-down approach that uses pathology to define disease. Precision medicine is a bottom-up approach that identifies predisease disorders using genetics, biomarkers, and modeling to prevent disease. This primer demonstrates the contrasting strengths and limitations of each paradigm and why precision medicine will eventually deliver on the promises.

## INTRODUCTION

Imagine a 30-year-old woman with 3 years of progressively worsening abdominal pain coming into your office in great distress because a computed tomography scan reveals inflammation and fibrosis in one of her digestive organs. She has 3 simple questions: “Why me?” “What is going to happen to me?” and “What treatment will stop this?” After a careful history, physical examination, and review of the computed tomography and other lab tests, she gets the brutally honest answer: “I don't know,” “I don't know,” and “I don't know.”

After billions of dollars and decades of work, our traditional approach to medical research into the early diagnosis and management of complex chronic diseases (CCD), including chronic inflammatory diseases (e.g., rheumatoid arthritis, inflammatory bowel disease, hepatitis/cirrhosis, chronic pancreatitis), has largely failed. We manage inflammation with super-expensive treatments, but we do not effectively address the underlying disorder. The size of this failure is profound because CCDs consume >90% of health care costs in the United States (1) and cause untold suffering in billions of people worldwide. We must do better!

There are 2 major science-based systems for diagnosing and managing diseases: Western medicine (allopathic medicine) and precision medicine (including personalized and individualized medicine). Western medicine is based on the premise that *one predominant and strong agent* causes disease in people who are otherwise *normal*. Precision medicine is the alternative system based on the premise that *one or more weak agents* cause disease in a person because one or more of their specialized cells are *abnormal.* Thus, the approach, methods, analysis, and results are expected to be very different—but not mutually exclusive.

## HOW MODERN WESTERN MEDICINE WORKS

### Approach to the patient

Modern Western medicine is a population-based, “top-down” approach to medicine. Disease diagnosis and treatment relies on traditional *clinicopathologic* definition and classification of disease. Signs and symptoms of disease lead to the collection of subjective and objective information and biological samples to identify the underlying etiology. If the evaluation does not identify a pathogen (e.g., microorganism) or cancer, then biomarkers (2) of the pathologic processes are used to make a “descriptive” diagnosis based on consensus criteria of disease features and pathologic severity (i.e., a syndrome of uncertain etiology such as inflammation in an organ without infection). The patient is then treated using evidence-based medicine (EBM).

### Approach determining disease etiology

The modern Western medicine paradigm is based largely on the germ theory of disease and the scientific method as highlighted in the medical education curriculum from the Flexner Report (1910) (3–5). The “scientific method” seeks to find the principal factor (i.e., a pathologic agent) causing a disease in a defined population following Koch's postulates (Table 1). It begins by assembling data, forming a hypothesis, and identifying the factor in the population of affected subjects that is *least likely* to be associated with the outcome by chance (e.g., *P* < 0.05). In some cases, the link between an agent and a disease was too complex to prove direct causality (i.e., cancer). In 1965, 9 “Bradford Hill Criteria” were proposed to link association with probability of causation (strength of association, consistency, specificity, temporality, biological gradient, plausibility, coherence, experiment, and analogy), although the actual disease *mechanisms* remained obscure (6). These criteria remain useful for public health, but do not determine *which* patient will develop a disease or how to target therapy.

**Table 1.**
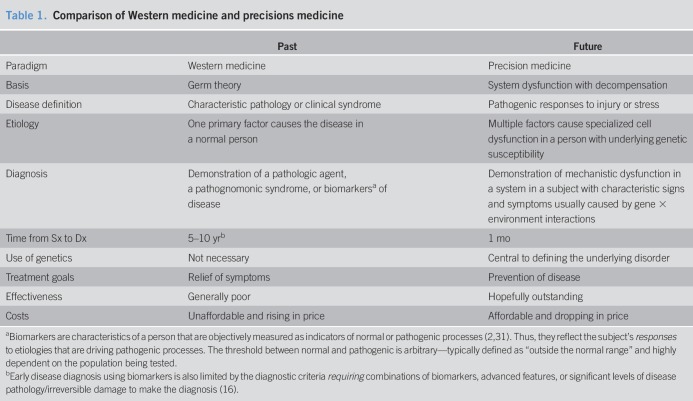
Comparison of Western medicine and precisions medicine

### Evidences

**Figure 1.**
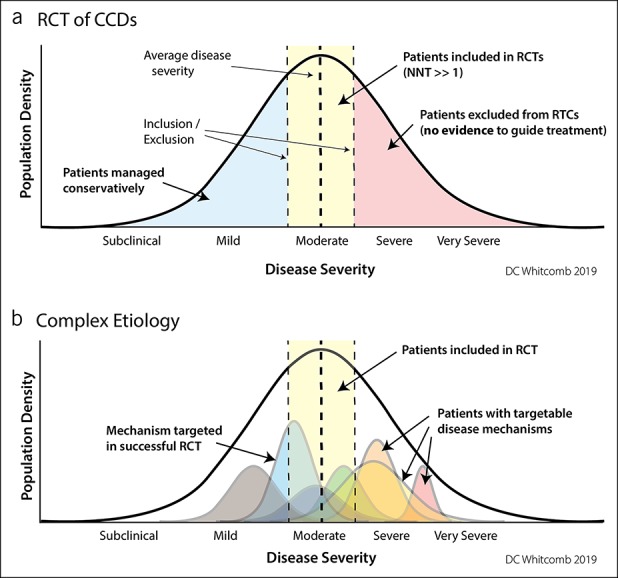
Therapeutic trials using clinicopathologic disease criteria. (**a**) Randomized clinical trials attempt to reduce heterogeneity by selecting the maximum number of patients with the least variability in disease features using inclusion–exclusion criteria. In CCDs, the treatment response is mixed with the NNT >>1. The patients with the highest burden of disease and in need of effective treatment are excluded from traditional clinical drug trials. (**b**) The same disease population seen as a function of multiple underlying disorders (colored curves) that may be a function of a single or multiple factors. A RCT targeting a low-severity mechanism (blue curve) will have “strong evidence” of effectiveness in the RCT, but will be of no value in more severe disease mechanisms (yellow, orange, and red curves). New approaches are needed to apply drug trials to mechanisms rather than common symptoms. CCD, complex chronic diseases; NNT, number needed to treat; RCT, randomized controlled trial.

Treatment selection and effectiveness is determined by clinical trials. To limit bias and minimize heterogeneity, investigators use randomized controlled trials (RCTs) by selecting patients with typical disease using detailed inclusion–exclusion criteria (Figure 1a). When trials are underpowered or conflicting results, then systematic reviews and meta-analyses are used to inform EBM.

### Paradigm-defined limitations

Western medicine works well for infectious, toxic, Mendelian genetic, and simple diseases, but it fails if the disease is complex. Complex can mean that *multiple underlying disorders* can alter the same biomarkers or result in the same pathologic process. It can mean that a disorder requires the *combined effect* of multiple factors where an independent factor is neither necessary nor sufficient to cause the disease. Complexity can also refer to a *highly variable clinical course* with unpredictable rates of progression, clinical features, or response to treatment. Figure 1b illustrates the challenges in designing a RCT for a CCD when randomization is based on descriptive definitions rather than disease mechanisms and why treatment targeting one mechanism results in a number needed to treat (NNT) for disease improvement is >1. The figure also shows why *no* EBM exists for patients outside inclusion criteria of RCTs (7), especially the most severe cases.

## HOW PRECISION MEDICINE WORKS

### Approach to patients

Precision medicine for CCDs is a cell dysfunction, “bottom-up” approach that seeks to provide *the right treatment to the right patient at the right dose at the right time* with expectation of better health at a lower cost. The goal is to determine the mechanisms causing an underlying *disorder* in an individual, symptomatic patient before the process leads to an irreversible chronic disease by managing the underlying disorder.

### Dysfunction → disorder → disease

A medical disorder indicates disruption of the normal functions of specialized cells resulting in abnormal signs, symptoms, biomarkers, or responses. The specialized cells are machines that are built and maintained and function through the action of proteins that are regulated through the cell's DNA in response to internal and external factors. In addition to the cell's normal and specialized function, it must respond to internal or external injury, toxins, or stresses. A disorder develops when the threshold for managing or adapting to the injury, toxin, or stress is exceeded, leading to a pathogenic response. The adaptive threshold can be markedly lower than normal if one or more key proteins within the machine are inherently dysfunctional (e.g., altered amino acid sequence) or dysregulated (e.g., expressed in the wrong place, at the wrong time, or in the wrong amount). This can make a person susceptible to a disorder and eventually disease under various environmental or metabolic conditions to which the average person easily adapts.

### Use of genetics

Genetic testing for precision medicine focuses on variants in the patient's genomic (inherited, germline) DNA, whereas precision medicine for cancer focuses on the tumor (i.e., precision therapeutics). It also differs from Mendelian genetics by considering multiple variants simultaneously, rather than limiting analysis to rare, highly pathogenic variants in a single gene as provided by traditional genetic reports that are nearly useless in complex disease management.

### Use of disease models

Interpretation of the impact of hundreds of potential genetic variants in a single patient requires highly structured, progressive disease models that define the effects of genetic variants on specific proteins within the context of active, specialized cells, within the structure and context of an organ. These models must be placed in the context of larger biological systems, with the influence of metabolic and environmental risk factors. Although the ability to completely integrate all relevant factors remains in the future, significant progress is being made in critical pieces of the puzzle for many CCDs including inflammatory bowel disease (8–10), liver diseases (11–13), and other noncancerous gastrointestinal diseases (14). However, this knowledge has not yet been integrated into patient-specific, dynamic, mechanistic models that predict disease etiology, progression, complications, and optimal interventions. In contrast, rapid progress is being made in precision medicine for recurrent acute and chronic pancreatitis. The simplicity of the organ (2 cell types that each have one primary function) (5) allows useful disease models to be built. Furthermore, the international pancreatology community is pushing the field forward by reaching consensus on a new *Mechanistic Definition* of chronic pancreatitis (15,16), progressive disease models (5,15), and use of consensus risk/etiology lists (17–19). From a clinical standpoint, consensus statements from authoritative groups that genetic testing is *medically necessary* as a part of the evaluation of recurrent acute pancreatitis (20,21) and chronic pancreatitis (22,23) mean that appropriate testing with a precision medicine report (genetics report plus clinical guidance for the individual patient) should be covered by reasonable health insurance plans.

### Diagnosis of medical disorders

**Figure 2.**
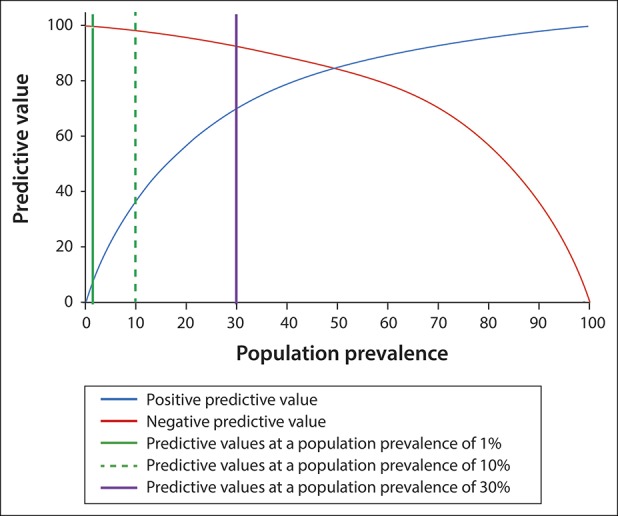
Effect of defining genetic risk factors in defined subpopulations to improve biomarker performance. In this example (a biomarker with a sensitivity of 85% and specificity of 85%), the identification of high-risk genetic risk factors moves a patient from a low-risk population (e.g., 1% prevalence) or patients with some disease symptoms (10% prevalence) to a subpopulation of patients with a high disease prevalence (e.g., 30%). Knowing the underlying mechanistic disorder through genetic analysis also adds specificity and also provides possible treatment targets.

Precision medicine focuses on diagnosing a disorder-causing signs and symptoms, often years before the disorder leads to an irreversible disease. The approach to diagnosis of a medical disorder in precision medicine includes (i) recognizing clinical signs, symptoms, or abnormal biomarkers, (ii) identifying pathogenic genetic variants linked to the disease, and (iii) testing for cell/system dysfunction. Based on these evidences, early treatment may be indicated. The advantage of a positive genetic test is that (i) it adds both specificity and accuracy to the interpretation of abnormal biomarkers (Figure 2), (ii) it limits the need for extensive and expensive traditional diagnostic testing, and (iii) it may dictate specific treatment years before traditional diagnoses can be made. Furthermore, it is anticipated that changes in lifestyle, environment, diet, or other inexpensive interventions may restore health and avoid the eventual cost of irreversible disease. An ounce of prevention is worth a pound of cure.

### Evaluating effectiveness

Because each patient is different, clinicopathology disease-defined RCTs are not expected to work in identifying treatments based on descriptive diagnoses without high NNTs (Figure 1). New approaches are needed such as N-of-1 trial design (24–26) or “basket” studies where a group of small adaptive RCTs focusing on targeting the disease mechanism are available for patients with mechanistic dysfunction—similar to many cancer studies targeting tumors (27–29). In the end, verifiable superiority of a precision medicine approach over “standard of care” is needed to practice EBM.

## LIMITATIONS OF THE PRECISION MEDICINE SYSTEM

### Too much complexity

Precision medicine disease dynamic, mechanistic disease models are difficult to develop. The popularity of data-driven “agnostic” models stems from the fact that they are much easier and can be useful in identifying principle disease drivers and dynamic networks. However, they are population-based approaches that, for individual patients, fail to identify the underlying etiologies, the likely outcomes, or the best therapies. The challenge in the future is integrating the top-down and bottom-up approaches within a single patient to provide new and effective solutions to manage human disease.

### Implementation

Moving precision medicine into practice continues to be impeded by multiple knowledge-based, value-based, acceptance, empowerment, and logistic barriers (30). It is also clear that modeling complex trait genetics is very complicated and physicians do not have the time or training to research every variable. The future requires development and implementation of new approaches for early diagnosis of pathogenic disorders and development of long-term management tools that are highly automated, highly accurate, and affordable. The future starts now!

## CONFLICTS OF INTEREST

**Guarantor of the article:** David C. Whitcomb, MD, PhD.

**Specific author contributions:** D.C.W. conceived of and wrote the article.

**Financial support:** This work was supported, in part, by NIH NIDDK DK108306.

**Potential competing interests:** D.C.W. is a consultant for AbbVie, Regeneron, and Ariel Precision Medicine and has equity in Ariel Precision Medicine.
